# Our experience in developing and operating the Airway Intervention Registry for Recurrent Respiratory Papillomatosis (AIR-RRP): national data collection [version 2; peer review: 2 approved]

**DOI:** 10.3310/nihropenres.13244.1

**Published:** 2023-01-12

**Authors:** Andrew Sims, Kim Keltie, Emma Belilios, Julie Burn, Liz Brown, Aaron Jackson, Steven Powell, Sue Jones, Adam Donne

**Affiliations:** 1 The Newcastle Upon Tyne Hospitals NHS Foundation Trust, Newcastle, NE7 7DN, UK; 2 Translational and Clinical Research Institute, Faculty of Medical Sciences, University of Newcastle upon Tyne, Newcastle, NE1 7RU, UK; 3 Population Health Sciences Institute, Faculty of Medical Sciences, University of Newcastle upon Tyne, NE1 7RU, UK; 4 Speech and Language Therapy, Manchester University NHS Foundation Trust, Manchester, M13 9WL, UK; 5 Alder Hey Children’s NHS Foundation Trust, Liverpool, L12 2AP, UK

**Keywords:** Recurrent respiratory papillomatosis (RRP) clinical registry development

## Abstract

Recurrent respiratory papillomatosis (RRP) is characterised by benign wart-like growths in the respiratory tract caused by the human papillomavirus (HPV). These warts vary in size and grow quickly, causing voice changes and airway obstruction. Whilst the condition is rare, RRP is more common and aggressive in children. There is currently no curative treatment for HPV, therefore RRP is managed by maintaining a safe airway and a serviceable voice by repeated surgery to remove the growths.

A lack of specific diagnostic codes prevents reliable case ascertainment of RRP from routine administrative databases such as Hospital Episode Statistics. In 2017 a cross-sectional survey identified 918 RRP patients in the UK, half of whom had received surgical intervention for RRP in the previous 12 months with 16 different interventions.

Randomised controlled trials for RRP interventions are difficult due to the rarity of the disease, variation in severity and progression and non-standard care across the NHS. Consequently, there is a lack of definitive efficacy and safety evidence. The only national guidance for RRP interventions is “Radiofrequency cold ablation for respiratory papillomatosis” (NICE IPG434, 2017) which recommended further data collection due to lack of evidence. However, due to the wide variation in RRP management across the NHS, clinical opinion favoured that any data collection should include a comparison of safety and efficacy of all RRP interventions in order to advise which improved patient outcomes and quality of life.

To address lack of evidence, and inform the future care of RRP patients, we developed a registry and used it to collect real-world data from patients receiving treatment for RRP in NHS hospitals across the UK. The purpose of this paper is to share lessons learned from this national data collection exercise to inform future clinical registry development.

## Introduction

Recurrent respiratory papillomatosis (RRP) is a condition characterised by benign wart-like growths in the respiratory tract, caused by the human papillomavirus (HPV)^1^. These warts vary in size and grow quickly, causing voice changes, chronic cough and airway obstruction^2^, which make it difficult to breathe, speak and lead a normal life. People with RRP are commonly misdiagnosed with asthma, croup, allergies, vocal nodules, or bronchitis, which delays diagnosis and treatment. Symptoms of RRP are managed by maintaining a safe airway and a serviceable voice by repeated surgery, usually under general anaesthesia, to remove the growths. The condition is relatively rare, but is more common and aggressive in children (1.8 per 100,000 adults, 4.3 per 100,000 children)^3^, and tends to recur after treatment. The results a survey of 283 ear, nose and throat (ENT) consultants in the UK, conducted by our research group recently, identified 918 RRP patients, of which 479 received at least one intervention in the previous 12 months^4^.

There is a lack of national guidance on how to manage the RRP condition. Controlled trials for this condition are difficult because RRP is rare, disease severity varies, it is difficult to prospectively determine the severe cases of RRP and there is no consensus exists on appropriate outcome measures. In 2012, the UK National Institute for Health and Care Excellence (NICE) issued national interventional procedure guidance which recommended the use of radiofrequency cold ablation for the treatment of RRP, under special arrangements for clinical governance, consent and audit or research^5^. This was due to a lack of safety and efficacy evidence (in both quantity and quality), around the use of this treatment. However, there is wide variation in the clinical management of RRP across the NHS, with the recent UK study with ENT consultants describing the use of 16 different RRP interventions. Due to general lack of evidence regarding efficacy of RRP interventions, long-term safety and RRP disease progression, the study team aimed to develop a national longitudinal data collection to inform the future care of RRP patients.

## Funding

With support from the Newcastle Joint Research Office (NJRO) we secured funding from the NIHR’s Research for Patient Benefit (RfPB) programme to develop a secure online database (PB-PG-0416-20037) to capture information from 400 RRP patients. This involved a two-stage application process with a maximum funding cap of £150,000. Contributors to the grant application included the study team (clinical lead: ENT surgeon, technical lead: Head of Northern Medical Physics and Clinical Engineering (NMPCE), computer scientist, research scientist), local research nurse, local IT/Information Governance, NJRO, YPAGne, a health economist, business development manager, and Research Design Service North East. Letters of support were gained from NIHR Clinical Research Network (CRN) North East and North Cumbria, and NICE.

The grant included funding for data collection centres to support time for data entry. This reflected the desire to capture RRP care across the NHS, including smaller hospitals without dedicated ENT research nurse support. We used the model non-commercial agreement (mNCA) as a template for individual site agreements at each participating data collection centre to facilitate the transfer of funds. This caused confusion at some centres who felt the length and complexity of the agreement was disproportionate to the amount of money involved (£73 per patient entered). Setting up and managing separate agreements across 48 data collection centres was also resource intensive. To date, many centres have not invoiced regularly.

Originally, funding was secured to capture data on paediatric RRP patients only (as RRP has higher incidence in children) but clinicians advised of the importance of capturing data from adult patients who have either contracted RRP as adults or who have had the condition since childhood. A successful application to ethics and funder was made to expand the registry to include adults.

## Public and patient involvement (PPI)

Due to higher prevalence in children, the research team engaged with the Young Persons Advisory Group – North East (YPAGne) during study design conceptualisation, prior to funding application. Feedback was incorporated into study design, the age-tailored patient information sheets, publicity to encourage recruitment and outputs of research to ensure the results were accessible to patients. YPAGne also recommended developing a study-specific patient website to update participants (and others) on study progress. Due to slow recruitment, and the expansion of the study to include adults, additional input was sought from the Newcastle Advising Patient Experience (APEX) and NIHR VOICE patient groups. An adult RRP patient and a parent of a child with RRP were recruited to the research study steering group. Early engagement with PPI groups positively influenced the study design, made the study more accessible to the target demographic and strengthened both ethics and grant applications. The study email address was added to the study website and sent to clinicians, patients and their families *via* posters in voice clinics across the UK (distributed on our behalf by the British Voice Association (BVA)). 18 people (patients or parents of children affected by RRP) contacted the team directly to request joining the study, which emphasised the importance of this study to people affected by RRP.

Additional publicity activities promoted the study website directly to patients. The BVA distributed leaflets at voice clinics and circulated a link to the study website to their membership (a mix of professionals and members of the public, all with an interest in promoting ‘voice’). The RRP data collection was also presented at the annual ‘Choice for Voice’ conference in September 2021. Further organisations agreed to post a link to the study website on their websites or Facebook pages: The Royal College of Speech and Language Therapists (RCSLT) published information in their Bulletin section, RRP patient support group, the RRP Foundation, shared information with their membership via their website and Facebook pages. The study was registered on Orphanet, a discussion thread was posted on Mumsnet (online parenting forum) and the study website was promoted in the NuTH research twitter feed.

## Ethics and information governance

Approval from the Health Research Authority (HRA) is not required for the establishment of research databases. However, to avoid the need for independent ethics review at each contributing centre, the study was submitted as a research database to the Integrated Research Application System (IRAS) to seek opinion from a Research Ethics Committee (REC). Favourable ethical opinion for the registry platform, the Airway Intervention Registry, was provided on 29^th^ December 2014 (IRAS ref: 164160). Centres agreeing to contribute data were recruited on an ongoing basis. Although not required for this project, when submitting as a research database the team obtained delegated authority from the REC to approve others to apply and use the data. We informed REC of new centres within annual reports.

Throughout data collection, we submitted four substantial amendments to REC. These were: SA1 - broadening inclusion of all RRP interventions including adjuvant therapies and inclusion of adults; SA2 - inclusion of patient invitation letter to support recruitment and changes to patient consent form to refer to UK Data Protection Bill 2018 in line with GDPR; SA3 - extended data collection; SA4 -additional datafields to capture COVID impact. There were also four non-substantial amendments: NSA1 - permitting retrospective consent; NSA2 - extended different data collection (not RRP); NSA3 - updating reference number on study documentation; NSA4 - extended data collection.

General understanding of research databases was lacking amongst participating centres. Most R&D departments continued to review and approve the study, and requested the form of documentation typically required for controlled trials (*e.g*. HRA letter of approval, delegation log). A further disadvantage of research databases is the maximum length of REC opinion is five years. To extend data collection, the research team had to submit an additional IRAS application (IRAS renewal: 288078). This required the study team to change all study documentation and formally notify data collection centres. This caused a substantial and unnecessary administrative burden as the purpose and method of data collection had not changed. In hindsight, submitting to IRAS as an observational study would have required individual review at each contributing centre (which most did, in any case), but would have caused less confusion during study set up and simplified study extensions.

To link information from multiple visits over time (potentially across multiple hospitals), the registry required patient identifiers including NHS number, date of birth, gender, and partial GP postcode. Patient information sheets, consent forms and assent forms were developed for parents/guardians of children under 5 years and for different age ranges (5–11 years, 12–15 years, 16+ years). An application was made to the Newcastle upon Tyne Hospitals NHSFT Caldicott Guardian for approval to collect the data.

## Trial registration

Following confirmation of funding, the research team prospectively registered the study (NCT03465280 on 14^th^ March 2018). Registration with ISRCTN was delayed; NIHR confirmed that exclusively observational studies were not eligible for free registration, and when the research team paid the registration fee the registration was completed (ISRCTN36100560 on 8^th^ May 2018).

## Registry specification and design

Steps involved in developing and running a clinical registry range from defining data fields and optimising data quality, through to encouraging participation and information governance. Invaluable advice for establishing and operating patient registries is available in the Agency for Healthcare Research and Quality (AHRQ) User’s Guide^6^. The purpose of the registry was to capture outcomes from interventions associated with RRP. Its design drew on our previous experience with procedural registries with linkage to routine data for cardiac and respiratory procedures^7–10^, with design adaptations acknowledging the chronic nature of RRP.

Involving clinicians in the registry design helped ensure that the sequence of data entry matched typical patient pathways. Data fields were defined in consultation with two ENT surgeons and one research nurse and included: demographic information, clinical history, patient and parent/guardian voice assessment questions, results from clinical examinations (including disease severity assessed by the Derkay score), histology results, details of RRP interventions (procedures and adjuvant therapies), post-procedural outcomes (including residual papillomas, complications, delay to discharge), and long-term outcomes (including voice assessments, endoscopic examination results, need for additional surgical intervention or adjuvant therapy, mortality).

The registry was designed for ENT surgeons, registrars, fellows, SLTs, research nurses and local data managers to enter data directly into the register, to avoid the need for central data entry and quality assurance. This required us to incorporate data field validation checks to improve data quality at the point of entry and minimise free text responses to avoid misinterpretation and complex free text mining during analysis. We developed draft data entry form specifications using Microsoft Word 2010 (open-source alternative: OpenOffice Writer). For each field, the specification defined its label, its format (date, number, free text, radio, multiple choice), its permitted values and whether it was mandatory. The form specifications and control flow diagrams underwent several rounds of external clinical review to ensure applicability across the NHS before handing over to the software developer.

The registry platform was created in house by NMPCE (JB) and hosted by the Newcastle upon Tyne Hospitals NHS Foundation Trust (NuTH). To protect patient data, an SSL security certificate was used to encrypt all information transmitted to and from the website. The RRP data collection was piloted for one week to test functionality and to gain feedback on ease of use from 10 ENT surgeons outside the direct research team before it officially opened on 1^st^ April 2018. Software development was undertaken using Zend Framework 2 (an open-source PHP framework^11^), which enabled expansion of the Airway Intervention Registry (AIR) to incorporate the RRP data collection. AIR is hosted on the Health and Social Care Network (HSCN) and only accessible *via* computers connected to the HSCN. Only clinical and research staff from NHS Trusts, with access approved by their Trust’s designated principal investigator, were issued with a password-protected account to view and enter data into the online registry. This level of access is required due to the collaborative nature of ENT and respiratory departments and the urgency with which some RRP patients require treatment. It also prevented data duplication. An advantage of in-house development was that content could be modified throughout data collection, *e.g.* COVID data fields were introduced in July 2020 to determine impact on RRP patients. The registry collected longitudinal data from recruited RRP patients, enabling new hospital visits to be recorded over time to capture relevant detail from related ENT and respiratory hospital visits (such as A&E attendance, outpatient clinic appointments, voice assessments, endoscopic examination results, surgical details of subsequent surgical interventions to remove papillomas and use of adjuvant therapies including therapy, dose and mode of administration).

A pseudonymised extract from the RRP data collection were linked to Hospital Episode Statistics (pseudonymised data from the Hospital Episode Statistics (HES) and the Office of National Statistics (ONS) mortality datasets to capture the additional outcomes of hospital resource usage, incidence of cancer and mortality. HES and ONS were supplied quarterly under DARS agreement DAR-NIC-17011-Z1B4J) Linkage was also used to verify a subset of registry fields (e.g., number of patients entered by each trust, dates of procedures, length of hospital stay), although routinely collected administrative data does not capture and cannot be used to verify clinically detailed fields (e.g., Derkay score, VHI for disease severity). To verify comorbidities and procedures captured in future clinical registries, we would recommend aligning these with relevant clinical coding systems (e.g., ICD for diagnoses coding and OPCS for procedure coding). Future registries for chronic diseases should considering adding data fields for each visit to capture intent (e.g., whether a procedure was planned, or triggered by deterioration in symptoms).

## Research study website

**Figure 1:**
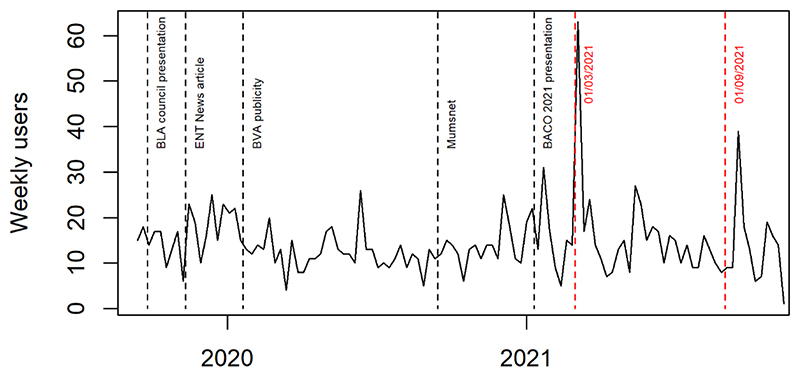
Number of users visiting study website each week.

Whilst the study website (*https://www.rrp.org.uk*) was developed to publicise study progress, it also included a secure portal for recruited patients (or their parents/carers for younger children) to submit voice assessment questionnaires whenever they felt there had been a change in their voice quality. Patients were provided with a unique identifier (eight-character alphanumeric) during the consent process. This enabled them to contribute quality of life data to the study in a secure but convenient manner (without requiring additional hospital visits). This was of particular benefit during COVID pandemic where elective hospital attendance reduced considerably; however, this relied on recruiting centres issuing identifiers and transcribing them to the registry for data-linkage purposes (which did not always occur). To comply with the information governance requirements of patient data being submitted over the internet, an approved supplier was contracted to host the study website. The study team were provided with a secure login to update patient and procedure numbers on the website which was conducted on a weekly basis. Weekly Google analytics reports allowed the team to monitor website activity, and gauge the success of publicity efforts (Figure 1). We also worked with the website hosts to improve the site’s visibility.

## Study management

The study team formed a project management group who met quarterly to discuss interim data, recruitment, safety outcomes and finances. Additionally, the registry platform had an overseeing Steering Group with membership including the study team, ENT surgeons, speech and language therapist, representation from professional societies (the British Association of Paediatric Otolaryngology, BAPO, ENTUK, and the British Laryngeal Association BLA), NICE, an adult patient with RRP and the parent of a child with RRP.

## Ongoing recruitment

**Figure 2:**
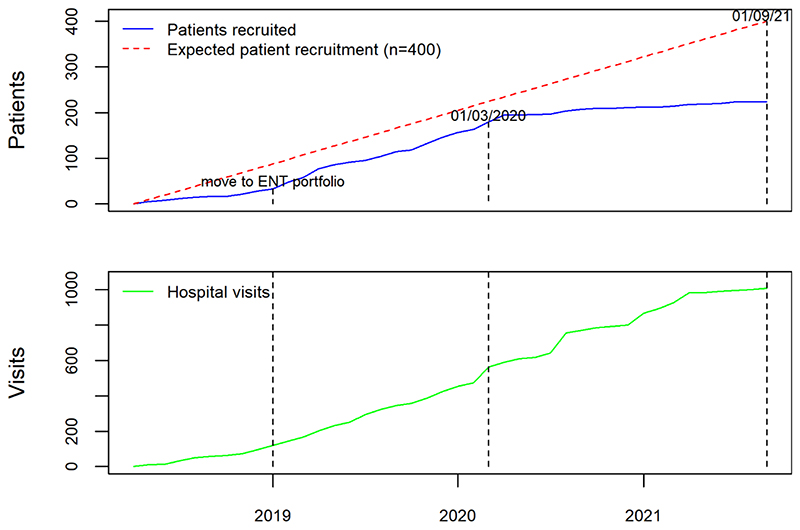
Number of patients and visits added to AIR RRP from 1^st^ April 2018 until 1^st^ September 2021.

Prior to the opening of the RRP data collection, and in order to ensure that the data collected were representative of practice across UK, the study team invited all acute NHS trusts with ENT departments to contribute as data collection centres. The study was publicised by three ENT professional societies (ENTUK, BLA, BAPO) who also signed a joint statement of support for the registry and encouraged their members to participate in the study. The study team liaised directly with clinicians and R&D departments at Centres to address issues with study set up and data entry. Regular newsletters were sent to all registered users, including personalised letters, which acknowledged their contribution to the registry for appraisal and re-validation purposes. The study team worked with local CRN (NENC) to publicise the study across the national network. When the study was expanded to include adults, the main speciality on the central portfolio management system (CPMS) changed from children to ENT, which resulted in a significant boost to recruitment (Figure 2). These efforts led to 48 NHS Trusts or Health Boards joining as formal data collection centres (39 in England, four in Scotland, four in Wales, and one in Northern Ireland).

The study website was presented at the NIHR portfolio development day. Participants were encouraged to discuss the website with their patients and to give them the unique ID needed to submit voice quality questionnaires *via* the portal. The website was commended in the BMA 2019 Patient Information awards.

As with many research studies, the pandemic has had a significant impact on patient recruitment due to the focus of research efforts and staff on COVID-related research. Due to this, NIHR approved an extension to data collection until 31^st^ August 2022. Of the 48 data collection centres, five have closed recruitment due to lack of capacity. However, data entry has remained open with centres contributing multiple hospital visits enabling longitudinal patient records capturing RRP outcomes over time. As of 16^th^ November 2021, we have collected information from 256 patients including 1057 hospital attendances, which represents the largest UK RRP data collection. Data has been contributed by ENT surgeons and respiratory physicians, registrars, fellows, research nurses, speech and language therapists, data managers, patients, and their families, making AIR RRP a comprehensive and collaborative ENT registry.

## Consent

The Airway Intervention Registry gained favourable ethical opinion as a research database on 29th December 2014 (IRAS for original application: 164160, renewal: 288078). Patients (or parents for participants under 16 years of age) are required to give written informed consent before their details can be entered into the online database. Age-appropriate Patient Information Sheets have been developed to assist with this.

## Data Availability

There are no underlying data associated with this article.

## REVISED: Amendments from Version 1

Changes made to the manuscript to address reviewer comments included additional text to describe who entered data into the registry, the specific outcomes recorded in the registry, additional references of other interventional procedure registries were added, and recommendations added to inform future registry development.

**Any further responses from the reviewers can be found at the end of the article**

## Plain English summary

Recurrent Respiratory Papillomatosis (RRP) causes wart-like growths in the airway, which make it difficult to breathe and speak. There is no known cure. Patients have their symptoms checked at hospital visits, and may need repeated surgical procedures to remove the growths and manage their symptoms. Different hospitals use a variety of different methods to manage RRP, and no one knows which methods work the best. We set up a registry to collect information from hospitals across the UK to understand more about which treatments for RRP work best for which patients. We learned a lot from the process of setting up and running this national data collection. The purpose of this paper is to share this learning to support the development of future clinical registries within the UK.

## Open Peer Review

**Current Peer Review Status:** ✓ ✓

Reviewer Report 14 February 2023

https://doi.org/10.3310/nihropenres.14488.r28952

**© 2023 Ongkasuwan J.** This is an open access peer review report distributed under the terms of the Creative Commons Attribution License, which permits unrestricted use, distribution, and reproduction in any medium, provided the original work is properly cited.

✓ **Julina Ongkasuwan**

^1^Department of Otolaryngology-Head and Neck Surgery, Baylor College of Medicine, Houston, TX, USA

^2^Texas Children's Hospital, Houston, TX, USA

The authors have done an admirable job addressing my concerns. I have no further edits at this time.

***Reviewer Expertise:*** Clinical research in voice, airway, and swallowing including RRP

**I confirm that I have read this submission and believe that I have an appropriate level of expertise to confirm that it is of an acceptable scientific standard.**

Reviewer Report 16 December 2022

https://doi.org/10.3310/nihropenres.14360.r28883

**© 2022 Ongkasuwan J.** This is an open access peer review report distributed under the terms of the Creative Commons Attribution License, which permits unrestricted use, distribution, and reproduction in any medium, provided the original work is properly cited.

? **Julina Ongkasuwan**^iD^

^1^Department of Otolaryngology-Head and Neck Surgery, Baylor College of Medicine, Houston, TX, USA

^2^Texas Children's Hospital, Houston, TX, USA

In this manuscript the authors outline their experience setting up a national database for collection of RRP data across the UK. There is no data to be analyzed nor results.

Many of the specific regulatory barriers the authors encountered appear to be specific the NHS and may not be generalizable to all countries. For example, I am rather surprised that ethical approval is not required for databases wherein patient specific identifiers are collected.

Is there any plan to continue to monitor RRP cases prospectively? To see changes in incidence with the HPV vaccine etc?

Public and patient involvement

○I applaud the authors for involving a patient and a parent. Was there any thought in using social media?

Ethics and governance

○The administrative hurdles appear to be very specific to the NHS. Were there other disease specific databases from other specialties upon which the RRP database could have been modeled?

Registry specification

○Please elaborate on "post-procedural outcomes and long-term outcomes." Does this mean surgical complications? Voice outcomes? Intersurgical intervals?

Research study website

○Direct patient upload of voice questionnaires has been very useful in other studies including, the initially North American Airway Collaborative.

**Is the work clearly and accurately presented and does it cite the current literature?**

Yes

**Is the study design appropriate and is the work technically sound?**

Yes

**Are sufficient details of methods and analysis provided to allow replication by others?**

Yes

**If applicable, is the statistical analysis and its interpretation appropriate?**

Not applicable

**Are all the source data underlying the results available to ensure full reproducibility?**

No source data required

**Are the conclusions drawn adequately supported by the results?**

Partly

***Reviewer Expertise:*** Clinical research in voice, airway, and swallowing including RRP

**I confirm that I have read this submission and believe that I have an appropriate level of expertise to confirm that it is of an acceptable scientific standard, however I have significant reservations, as outlined above.**

Reviewer Report 11 April 2022

https://doi.org/10.3310/nihropenres.14360.r28532

**© 2022 Best S.** This is an open access peer review report distributed under the terms of the Creative Commons Attribution License, which permits unrestricted use, distribution, and reproduction in any medium, provided the original work is properly cited.

✓ **Simon R. Best**

Department of Otolaryngology-Head and Neck Surgery, Johns Hopkins University, Baltimore, MD, USA

I commend the authors for their work and the massive undertaking of starting a nation-wide registry for RRP. This rare disease certainly suffers from a lack of vigorous research and the inability of any single clinical site to assemble a large enough cohort is one of the problems.

The authors of this study have attempted to solve this problem by using the entire UK as a source of RRP patients. Attempting a registry at this scale will inevitably have a series of roadblocks and hurdles to even implement in the first place, and then once up and running will have a whole different set of challenges. This manuscript describes some of the problems that were encountered and suggests potential solutions, to help other groups in the future. The series of steps that it took to implement the registry are well-described but there are few areas where clarification is requested:

1. The number of patients registered is not clear. The abstract states 918 patients, but the manuscript states 256 patients, and the recruitment figure matches this later number.
2. It is clear from the manuscript that patients were able to directly enter quality of life data using their unique ID. However, it was less clear from the descriptions in the manuscript how other data was entered. Were enrolled sites entering their own data, or sending it to a central site for quality assurance before entering. It certainly would be possible either way, and the relative benefits and downsides could be discussed.
3. The authors should discuss in more detail what fields they think have provided the most robust data and what fields were of low-yield or did not provide useful information. I certainly expect that other manuscripts are in the works for this registry, but having some sense of what data is more or less reliable will help other groups in their planning.

Overall, this is a very impressive undertaking and an excellent manuscript that describes a truly herculean task.

**Is the work clearly and accurately presented and does it cite the current literature?**

Yes

**Is the study design appropriate and is the work technically sound?**

Yes

**Are sufficient details of methods and analysis provided to allow replication by others?**

Yes

**If applicable, is the statistical analysis and its interpretation appropriate?**

Partly

**Are all the source data underlying the results available to ensure full reproducibility?**

No source data required

**Are the conclusions drawn adequately supported by the results?**

Yes

***Reviewer Expertise:*** Voice disorders, Recurrent Respiratory Papillomatosis

**I confirm that I have read this submission and believe that I have an appropriate level of expertise to confirm that it is of an acceptable scientific standard.**

**Comments on this article**

**Version 1**

Author Response 22 Dec 2022

**Kim Keltie**, The Newcastle Upon Tyne Hospitals NHS Foundation Trust, Newcastle, UK Response to reviewer 2 (addressed in version uploaded on 21/12/2022).

Reviewer 2

In this manuscript the authors outline their experience setting up a national database for collection of RRP data across the UK. There is no data to be analysed nor results.

Many of the specific regulatory barriers the authors encountered appear to be specific the NHS and may not be generalizable to all countries. For example, I am rather surprised that ethical approval is not required for databases wherein patient specific identifiers are collected.

Is there any plan to continue to monitor RRP cases prospectively? To see changes in incidence with the HPV vaccine etc?

**Response:**

**Results from the registry have been written up in a separate manuscript and will be submitted to an ENT journal.**

**A hyperlink has been added the the “Ethics and information governance” subsection, to support that establishment of research databases do not require HRA approval within the NHS.**

**An additional sentence has been added to the “Registry specification and design” subsection to highlight that the registry collected longitudinal data from recruited RRP patients (adult and children) including data from outpatient appointments, A&E attendance, voice assessments, endoscopic examination results, details of subsequent surgical interventions and adjuvant therapies.**

Public and patient involvement

- I applaud the authors for involving a patient and a parent. Was there any thought in using social media?

**Response: Additional text moved into PPI section (was previously present in the ongoing recruitment section). This includes discussion of how the publicity incorporated social media.**

Ethics and governance

- The administrative hurdles appear to be very specific to the NHS. Were there other disease specific databases from other specialties upon which the RRP database could have been modelled?

**Response: The hurdles were specific to the UK (rather than to the NHS) because that was its setting. We based the register design on previous experience with surgical registries linked to routine data. We have added material to the first paragraph of the registry specification and design section to explain this point.**

Registry specification

- Please elaborate on "post-procedural outcomes and long-term outcomes." Does this mean surgical complications? Voice outcomes? Inter-surgical intervals?

**Response: Additional outcomes added.**

Research study website

- Direct patient upload of voice questionnaires has been very useful in other studies including, the initially North American Airway Collaborative.

**Response: Thank you for your comment. No change required.**

**Kim Keltie**, The Newcastle Upon Tyne Hospitals NHS Foundation Trust, Newcastle, UK Response to reviewer 1 (addressed in version uploaded on 21/12/2022).

Reviewer 1:

I commend the authors for their work and the massive undertaking of starting a nation-wide registry for RRP. This rare disease certainly suffers from a lack of vigorous research and the inability of any single clinical site to assemble a large enough cohort is one of the problems.

The authors of this study have attempted to solve this problem by using the entire UK as a source of RRP patients. Attempting a registry at this scale will inevitably have a series of roadblocks and hurdles to even implement in the first place, and then once up and running will have a whole different set of challenges. This manuscript describes some of the problems that were encountered and suggests potential solutions, to help other groups in the future. The series of steps that it took to implement the registry are well-described but there are few areas where clarification is requested:

1. The number of patients registered is not clear. The abstract states 918 patients, but the manuscript states 256 patients, and the recruitment figure matches this later number.

**Response: 918 RRP patients in the UK were identified from a previous survey published in 2017. The registry described in this paper includes (as of August 2022) clinical and longitudinal data from 256 patients in the UK. We have amended the abstract text to clarify the distinction between the two.**

1. It is clear from the manuscript that patients were able to directly enter quality of life data using their unique ID. However, it was less clear from the descriptions in the manuscript how other data was entered. Were enrolled sites entering their own data, or sending it to a central site for quality assurance before entering. It certainly would be possible either way, and the relative benefits and downsides could be discussed.

**Response: Data was entered directly into the registry from ENT surgeons, registrars, fellows, SLTs, research nurses and data managers. We have clarified this in the registry specification and design section. In the third paragraph of that section, we have described the design process used to ensure that data entry forms matched clinician workflows, that entry into each data field was subject to pattern and logical validation and clarified that such steps are necessary for registries with local data entry.**

1. The authors should discuss in more detail what fields they think have provided the most robust data and what fields were of low-yield or did not provide useful information. I certainly expect that other manuscripts are in the works for this registry, but having some sense of what data is more or less reliable will help other groups in their planning.

**Response: We have added a paragraph at the end of the “Registry specification and design” section to describe data fields which could be verified with routine administrative data, and have included a recommendation which may benefit the quality of data collected in future**

**interventional registries.**

Overall, this is a very impressive undertaking and an excellent manuscript that describes a truly herculean task.
