# Supplementary material for: Our experience in developing and operating the Airway Intervention Registry for Recurrent Respiratory Papillomatosis (AIR-RRP): national data collection
Source: NIHR Open Res. 2023 Jan 12;2:22. Originally published 2022 Feb 24. [Version 2] doi: 10.3310/nihropenres.13244.2 (PMC7614251; doi:10.3310/nihropenres.13244.2)
Supplement: Peer review history

# Reviewer report: Reviewer response for version 2

**DOI:** 10.3310/nihropenres.14488.r28952

**Published:** 2023-02-14

**Authors:** Julina Ongkasuwan

**Affiliations:** 1 Texas Children's Hospital, Houston, TX, USA; 2 Department of Otolaryngology-Head and Neck Surgery, Baylor College of Medicine, Houston, TX, USA

The authors have done an admirable job addressing my concerns. I have no further edits at this time.

Is the work clearly and accurately presented and does it cite the current literature?

Yes

If applicable, is the statistical analysis and its interpretation appropriate?

Not applicable

Are all the source data underlying the results available to ensure full reproducibility?

No source data required

Is the study design appropriate and is the work technically sound?

Yes

Are the conclusions drawn adequately supported by the results?

Partly

Are sufficient details of methods and analysis provided to allow replication by others?

Yes

Reviewer Expertise:

Clinical research in voice, airway, and swallowing including RRP

I confirm that I have read this submission and believe that I have an appropriate level of expertise to confirm that it is of an acceptable scientific standard.

# Reviewer report: Reviewer response for version 1

**DOI:** 10.3310/nihropenres.14360.r28883

**Published:** 2022-12-16

**Authors:** Julina Ongkasuwan

**Affiliations:** 1 Texas Children's Hospital, Houston, TX, USA; 2 Department of Otolaryngology-Head and Neck Surgery, Baylor College of Medicine, Houston, TX, USA

In this manuscript the authors outline their experience setting up a national database for collection of RRP data across the UK. There is no data to be analyzed nor results.

 Many of the specific regulatory barriers the authors encountered appear to be specific the NHS and may not be generalizable to all countries. For example, I am rather surprised that ethical approval is not required for databases wherein patient specific identifiers are collected.

 Is there any plan to continue to monitor RRP cases prospectively? To see changes in incidence with the HPV vaccine etc?

 Public and patient involvement 

- I applaud the authors for involving a patient and a parent. Was there any thought in using social media?

Ethics and governance 

- The administrative hurdles appear to be very specific to the NHS. Were there other disease specific databases from other specialties upon which the RRP database could have been modeled?

Registry specification

- Please elaborate on "post-procedural outcomes and long-term outcomes." Does this mean surgical complications? Voice outcomes? Intersurgical intervals?

Research study website

- Direct patient upload of voice questionnaires has been very useful in other studies including, the initially North American Airway Collaborative. 

Is the work clearly and accurately presented and does it cite the current literature?

Yes

If applicable, is the statistical analysis and its interpretation appropriate?

Not applicable

Are all the source data underlying the results available to ensure full reproducibility?

No source data required

Is the study design appropriate and is the work technically sound?

Yes

Are the conclusions drawn adequately supported by the results?

Partly

Are sufficient details of methods and analysis provided to allow replication by others?

Yes

Reviewer Expertise:

Clinical research in voice, airway, and swallowing including RRP

I confirm that I have read this submission and believe that I have an appropriate level of expertise to confirm that it is of an acceptable scientific standard, however I have significant reservations, as outlined above.

# Reviewer report: Reviewer response for version 1

**DOI:** 10.3310/nihropenres.14360.r28532

**Published:** 2022-04-11

**Authors:** Simon R. Best

**Affiliations:** 1 Department of Otolaryngology-Head and Neck Surgery, Johns Hopkins University, Baltimore, MD, USA

I commend the authors for their work and the massive undertaking of starting a nation-wide registry for RRP. This rare disease certainly suffers from a lack of vigorous research and the inability of any single clinical site to assemble a large enough cohort is one of the problems.

 The authors of this study have attempted to solve this problem by using the entire UK as a source of RRP patients. Attempting a registry at this scale will inevitably have a series of roadblocks and hurdles to even implement in the first place, and then once up and running will have a whole different set of challenges. This manuscript describes some of the problems that were encountered and suggests potential solutions, to help other groups in the future. The series of steps that it took to implement the registry are well-described but there are few areas where clarification is requested:

1. The number of patients registered is not clear. The abstract states 918 patients, but the manuscript states 256 patients, and the recruitment figure matches this later number.
2. It is clear from the manuscript that patients were able to directly enter quality of life data using their unique ID. However, it was less clear from the descriptions in the manuscript how other data was entered. Were enrolled sites entering their own data, or sending it to a central site for quality assurance before entering. It certainly would be possible either way, and the relative benefits and downsides could be discussed.
3. The authors should discuss in more detail what fields they think have provided the most robust data and what fields were of low-yield or did not provide useful information. I certainly expect that other manuscripts are in the works for this registry, but having some sense of what data is more or less reliable will help other groups in their planning.

Overall, this is a very impressive undertaking and an excellent manuscript that describes a truly herculean task.

Is the work clearly and accurately presented and does it cite the current literature?

Yes

If applicable, is the statistical analysis and its interpretation appropriate?

Partly

Are all the source data underlying the results available to ensure full reproducibility?

No source data required

Is the study design appropriate and is the work technically sound?

Yes

Are the conclusions drawn adequately supported by the results?

Yes

Are sufficient details of methods and analysis provided to allow replication by others?

Yes

Reviewer Expertise:

Voice disorders, Recurrent Respiratory Papillomatosis

I confirm that I have read this submission and believe that I have an appropriate level of expertise to confirm that it is of an acceptable scientific standard.
